# The Problems of Modern Psychiatry—II

**Published:** 1911-11-18

**Authors:** Thomas Clouston

**Affiliations:** late Physician-Superintendent Royal Asylum, Edinburgh, and Lecturer on Mental Diseases, University of Edinburgh, etc.


### THE PROBLEMS OF MODERN PSYCHIATRY—II.

An Introductory Lecture to the Class of Mental Diseases, University of Edinburgh.

By Sir THOMAS CLOUSTON, M.D., LL.D., late Physician-Superintendent Royal Asylum, Edinburgh, and Lecturer on Mental Diseases, University of Edinburgh, etc.

The late Mr. F. Meyers formed the theory that lying at the back of conscious memories, feelings and acts there is a "subliminal consciousness" which covers a far vaster region than the conscious life of the individual. I explain the phenomena of this "subliminal consciousness" by the fact that there is impressed on the brain cells a molecular change by every impression received by the special senses to which attention is paid, by every mental feeling and thought and imagination, and that this change enables the acts of memory to be performed. When by association of things or ideas the former periods of life are suggested, we have in those molecular changes an apparatus by which the former mental experiences are brought before consciousness again. There is, in fact, far more latent in the brain cells than is ever present to consciousness. This explains also the unconscious cerebration of dreams. I would say about this "subliminal mind" that a "consciousness" that is not subjectively conscious is—as Mercier says—a "contradiction in terms."

It is certain that the development of the brain cells from birth to five-and-twenty is dependent on the continual stream of in-coming stimuli of various kinds, from the common sensory nerves, from the special sense organs, and from the viscera, to which they are being continually subjected during the waking life of the individual, "no senses, no intelligence." When those stimuli are not received, as, for instance, in children who are deaf and dumb and blind, the mind remains in a state resembling idiocy. It follows from this that if those stimuli are of the wrong sort, the mental action becomes perverted and so insanity may occur.

All stimuli produce reactions mental or bodily, and commonly both, in a healthy brain. The capacity of normal reaction is one of its most important functions. It may be exaggerated, lessened, or perverted, with fatal results to normal mind-working.

### Solidarity.

It is not surprising, looking to the mere anatomy of the brain, that it has what may be called a solidarity of action to an extraordinary degree. This characteristic of the brain must never be lost sight of in practical psychiatry. No nervous symptom referable to the brain can be neglected, because its morbid influence may spread. There is also a nutritive solidarity between the same points in the two hemispheres of the brain—a localised bi-symmetry and localised hæmorrhages are frequently found in the same places in the two hemispheres. So when we have a mental disturbance originating in the mental cortex we have very commonly indeed a spreading of the morbid condition to the sensory centres, the motor centres, and the nutritive centres of the brain, so causing marked bodily symptoms in a really mental case.

There is another quality of the brain which it is of great importance to remember in psychiatry, namely, its resistiveness. This quality enables it to suffer intense mental and bodily stress without being upset in its main function. We all know the amount of alcoholic poison, for instance, which the brain cortex will stand with almost impunity in some people. The brain will also tolerate traumatic and surgical injuries in a wonderful manner. One of the most simple examples of the brain's resistiveness is shown by the fact that in death from starvation every organ and other tissue of the body loses weight, some of them enormously, except the brain, which remains almost intact as regards its weight when every other tissue has, as it were, been starved to death. Without this innate power of resistiveness it is certain that an organ of such extraordinary complexity and infinite delicacy of structure and arrangement would suffer injury and disease from the causes operating in human beings to a much larger extent than it does.

### Sleep.

There is one function of the higher brain-cell of all-pervading importance in the practice of psychiatry; that is the sleep function. In regard to the times of its occurrence the sleep function is the most marked example of that great and all-pervading nervous quality of *periodicity of action.* The law of periodicity probably pervades all life down to the lowest uni-cellular organisms, if we had any means of estimating it there. We find the most complete illustration of the law of periodicity in the mental cell—for two-thirds of the day in early childhood, for half the day later on, and for a third of the twenty-four hours all through life it suspends the mental action, either completely or almost entirely. During that time mind does not exist; the cell simply ceases its katabolic action and devotes itself entirely to the building-up of its own structure—to its anabolic action. No form of insanity is so remarkable or unexplainable as the sleep function. Insomnia is perhaps the most common of all the preliminary symptoms of mental disease, occurring often for long periods before any mental symptoms have appeared. The re-establishment of the periodic suspension of mind in sleep is the very highest aim of the doctor who treats many cases of insanity. If we could devise a method of securing sound natural sleep to come on at the proper periodic intervals, I am satisfied we could prevent the occurrence of mental disease in one-third of the cases and cure another third in half the time we are now able to do.

There is a difference between any kind of drug sleep and natural sleep, not in favour of the former.

### Toxins and the Selective Power of the Brain and its Different Parts.

The brain cells are specially liable to be affected in their mental action by toxins of various kinds from within and without the body, endogenous and exogenous. The effects of such poisons as alcohol, opium, and Indian hemp, as well as syphilis, have been long known, marked, and studied with great care. The various conditions of delirium from the continued fevers have also naturally attracted attention for a long time, but that the conditions of mental depression, exaltation, delusion, and hallucination should be directly caused by toxic action from within the body has been realised fully only for the last few years. This view of the ætiology of many mental diseases has been greatly accentuated by the study of myxœdema, Graves' disease, and cretinism. Certain cases of acute confusional mania, some cases of melancholic depression, hallucinations, and especially all cases of general paralysis, are now regarded as toxic in origin. The sources of the toxin may be from the intestines, from the kidneys, from the blood, from the thyroid, parathyroid, and pituitary glands, or even as the result of perverted bio-chemical action in the cells themselves. The effects of different toxins may be to irritate, to depress, to pervert, or to paralyse mental action. But the different functional areas of the brain cells have an extraordinary selective power in regard to certain poisons, while others have an equally resistive power against them. We all know what happens as the result of alcoholic excess on the cortical cells. In one man the speech will be affected, in another the power of locomotion, in another the mental action, and looking at the various mental faculties, it is as surprising as it is puzzling that in one man we will find an exalted state of mind, in another depression, in a third mere confusion, and so forth. The exact bio-chemical action of those cortical poisons is not as yet known. There is a risk of that toxæmia, too, which results from interference with the drainage system of the brain. Many pathological hæmic conditions are both the causes and the results of mental disease. In considering the toxic action of the brain, the question has always to be asked: "Are they causes or symptoms, and if symptoms, do they aggravate the pathological disturbance?" We know that in certain conditions of morbid mental excitement or depression, the digestion, the action of the bowels, the skin, and the heart may be affected secondarily through disturbances in the cortical centres where these organs and functions are represented. It is quite certain that if we knew precisely the bio-chemistry of the brain cell in action we should discover that both in the anabolic and katabolic processes going on within it the molecular and chemical conditions would change as the activity of the cell changed. When a man is joyous, thinking keenly, and acting vigorously, it has been demonstrated that the temperature is greater over the mental regions of the cortex, and that the capillaries are then more congested. Certain poisons generally produce the same sort of mental action; for instance, alcohol increases the feeling of well-being and heightens the social instincts, atropine and cocaine will excite the visual centres and thus produce hallucinations, while antimony will produce a sense of misery and lethargy.

### What Constitutes Mental Health and Mental Disease?

The most recent Italian School would reduce mental diseases to three divisions:—

1. The Congenital.
2. The Traumatic.
3. The Toxic—comprising all the ordinary insanities.

There is a preliminary question which the student of psychiatry has to answer and that is: "What constitutes mental health?" This does not admit of being answered categorically. Mental health passes into mental disease most commonly in a gradual way, as light passes into darkness; there is a mental twilight, a borderland in which it is impossible to say whether the patient is mentally ill or not. You cannot say in the case of any individual whether he is in health or not till you know the mental condition which is normal to him. To be in a condition of mental disease implies a distinct difference from the normal state. It is always well for a man who undergoes such changes mentally to consult his doctor, and it is always well for the doctor not to make too light of such a change, because treatment is usually far more effectual in that borderland stage than it is when the symptoms have been fully developed. The best test of mental health is when a man feels a conscious sense of organic well-being, of *bien-être*, although it is to be admitted that many persons go through life with more or less of a sense of ill-being all the time, and are not on that account to be regarded as insane.

Another great test of whether a man is in mental health is this—how do his brain cells respond to the innumerable stimuli, mental and bodily, to which they are exposed every hour of his waking life? A normal response to such stimuli is the essential quality of a sane brain cell. Such stimuli, if the brain is in a normal condition, are received, diffused, selected, and transformed in accordance with certain laws in the brain cortex. If a man, for instance, is so near the fire that his flesh is in danger of being burned, and does not go away from it, he is in a state of mental unhealth. If he is told that his house is on fire, and does not take measures to call the fire-engine, he is in a morbid state. If he hears that his child has a cold, and goes and orders its coffin, he is in a state of mental unsoundness.

When we consider how many and how various those stimuli are, the wonder is that his brain cells continue to react normally during the whole life of a healthy man. Sensations of touch, vision, sounds, tastes, smells, the muscular sense, visceral action, thoughts, feelings, memory, everything in fact that is capable of producing a molecular change in the cell, may be the cause of disordered action in that cell. The stimuli need not necessarily be from the periphery to the brain; they may also take place from the brain down on to the nerve-endings in the various organs. To be in health, in short, implies a normal reactiveness to nerve stimuli of all kinds.

*(To be continued.)*

